# Impaired NLRP3 inflammasome signaling diverts pyroptotic to apoptotic caspase activation in macrophages

**DOI:** 10.3389/fimmu.2025.1631152

**Published:** 2025-10-24

**Authors:** Ying-qing Gan, Yuan-wen Cai, Xiu-wen Liang, Ling-Zhi Wang, Fu-li Shi, Nuo Sun, Ya-ping Li, Rong Xu, Bo Hu, Qing-bing Zha, Xian-hui He, Tak-sui Wong, Jin-hua Li, Dong-yun Ouyang

**Affiliations:** ^1^ Department of Immunology and Microbiology, College of Life Science and Technology, Jinan University, Guangzhou, China; ^2^ Guangdong Provincial Key Laboratory of Spine and Spinal Cord Reconstruction, the Fifth Affiliated Hospital (Heyuan Shenhe People’s Hospital), Jinan University, Heyuan, China; ^3^ Center of Reproductive Medicine, The First Affiliated Hospital of Jinan University, Guangzhou, China; ^4^ Department of Nephrology, The First Affiliated Hospital of Jinan University, Guangzhou, China; ^5^ Division of Nephrology, Department of Medicine, The First Affiliated Hospital of Jinan University Chaoshan Hospital, Chaozhou, China; ^6^ Department of Ultrasound, The First Affiliated Hospital of Jinan University, Guangzhou, China; ^7^ Department of Ultrasound, The Fifth Affiliated Hospital of Jinan University (Heyuan Shenhe People’s Hospital), Heyuan, China

**Keywords:** NLRP3 inflammasome, apoptosome, caspase-8, LPS tolerance, pyroptosis, apoptosis

## Abstract

NLRP3 (NLR family pyrin domain-containing 3) inflammasome is a first line of defense of innate immunity, mediating caspase-1-dependent pyroptosis and cytokine release upon danger signaling. Intervention of NLRP3 innate surveillance may cause defects in this signaling pathway, while the host has evolved alternative ways to combat such intervention. Yet it remains incompletely understood whether NLRP3 sensing of danger signaling can divert pyroptosis to other forms of cell death in circumstances of impaired NLRP3 signaling. In this study, we adopted two macrophage models (delayed delivery of triggering signaling and caspase-1 deficiency) to mimic defects in NLRP3 signaling to address this issue. We found that the NLRP3/ASC platform preferentially recruited caspase-1 rather than caspase-8 in lipopolysaccharide (LPS)-primed macrophages timely triggered with nigericin. However, when the triggering signal (nigericin) was delayed, the recruitment diverted to caspase-8, leading to apoptotic caspase activation. Furthermore, in caspase-1-deficient macrophages, nigericin triggering diverted NLRP3-ASC-caspase-1-driven pyroptosis to caspase-8/-9/-3 activation and GSDME-mediated secondary necrosis. Unexpectedly, VX-765 (a caspase-1 inhibitor) exhibited a pan-caspase inhibitor-like effect, suppressing caspase-8/-9/-3 activation and GSDME cleavage in a dose-dependent manner. Mitochondrial damage was observed in both WT and caspase-1-deficient cells upon nigericin stimulation, suggesting mitochondrial injury being an upstream event in this process. Collectively, our data indicate that NLRP3 inflammasome is poised to divert pyroptotic to apoptotic caspase activation for combating danger signaling when conventional pathway is impaired, highlighting a complex interaction between various forms of cell death pathways.

## 1 Introduction

The innate immune system is the first line of defense against various pathogenic infections or tissue damage. A central mechanism of this defense is the activation of various inflammasomes (1, 2). Specifically, the NLRP3 inflammasome, which is composed of NLRP3, ASC, and caspase-1, among other members, is tightly regulated by multiple signaling pathways. First, the expression of NLRP3 and pro-IL-1β is induced by pathogen-associated molecular patterns (PAMPs) or damage associated molecular patterns (DAMPs) such as lipopolysaccharide (LPS), while pro-caspase-1 and ASC (apoptosis-associated speck-like protein containing a CARD) are expressed constitutively (without the need of danger signal stimulation). As an adaptor, ASC binds NLRP3 via PYD-PYD domain interactions, and similarly interacts with caspase-1 through CARD-CARD domains. On the NLRP3 inflammasome or other forms of inflammasomes, caspase-1 undergoes autocleavage and activation, which in turn processes pro-IL-1β and pro-IL-18 into their mature forms and cleaves gasdermin D (GSDMD) to generate its N-terminal fragment (GSDMD-NT). GSDMD-NT fragment then oligomerizes on the cell membrane to form pores, facilitating the release of mature IL-1β, IL-18 and other DAMPs, and ultimately leading to pyroptotic cell death (1). These cytokines and DAMPs enhance immune responses by bridging innate and adaptive immunity, thereby promoting pathogen clearance (2, 3).

While hosts have evolved inflammasomes to sense and combat pathogens, viruses and other pathogens develop evasion strategies. For instance, Zika virus nonstructural protein NS3 inhibits cellular NLRP3 inflammasome activation by disrupting ASC oligomerization (4). Some viruses encode caspase-1 inhibitory proteins, such as SARS-CoV-2 NSP1 and NSP13 (5), or proteases (*e.g*., by *Enterovirus 71*) that directly cleave caspase-1 (6). In certain infections (e.g., *Salmonella*), caspase-1 and -8 can be independently activated, with caspase-8 activation being dependent on NLRC4 and ASC (7). Notably, NLRC4 activators or other danger signals may induce ASC-caspase-8-dependent apoptosis when caspase-1 is deficient (8–10). However, the mechanisms underlying this switch from caspase-1 to caspase-8 activation under impaired inflammasome signaling remain unclear.

Unlike acute infection, chronic one may induce immune tolerance, rendering immune cells insensitive to danger signals (11). Notably, classical monocytes isolated from Crohn’s disease patients exhibit “exhaustion”, characterized by reduced IL-1β release upon LPS stimulation, linking LPS tolerance to chronic inflammatory disorders (12). Prolonged exposure to endotoxin reprograms macrophages into a tolerant state, altering their metabolism (*e.g.*, respiration and secretome) (13, 14). On a systemic level, elevated circulating endotoxin can stimulate host leukocytes into a low-grade “memory” state, contributing to chronic diseases including atherosclerosis, diabetes, and Parkinson’s disease (15). Repeated LPS stimulation induces selective immunosuppression in lungs, reducing susceptibility to bacterial pneumonia but without impairing neutrophil responses (16). However, whether NLRP3 activators can trigger caspase-1 or caspase-8 activation in LPS-tolerant macrophages is unclear and warrants investigation.

In this study, we explored NLRP3/ASC signaling outcomes in macrophages with impaired inflammasome pathways by using two macrophage models (delayed triggering of NLRP3 and caspase-1 deficiency). Our results showed that the NLRP3/ASC platform preferentially recruited caspase-1 upon timely activation but switches to caspase-8/-3 under caspase-1 deficiency or delayed triggering (e.g., nigericin stimulation). In LPS-primed wild-type (WT) macrophages, ATP/nigericin activated canonical NLRP3-ASC-caspase-1 inflammasomes, leading to pyroptosis. In caspase-1-deficient cells, however, the same stimuli triggered caspase-8/-9/-3 activation, leading to GSDME-mediated secondary necrosis. Our data reveal that the NLRP3/ASC platform can redirect the activation of pyroptotic caspase-1 to apoptotic caspases, highlighting an alternative way for innate immune cells to cope with danger signaling.

## 2 Materials and methods

### 2.1 Reagents and antibodies

Belnacasan (VX-765, S2228, Purity: 99.99%) was obtained from Selleck Chemical (Houston, TX, USA), Belnacasan powder was dissolved in dimethyl sulfoxide (DMSO) at 20 mM and stored at −20°C. Propidium iodide (PI, P4170), Hoechst 33342 (B2261), CF568-conjugated goat-anti-rabbit IgG (SAB4600084), CF488-conjugated goat-anti-mouse IgG (SAB4600237), lipopolysaccharide (LPS, *Escherichia coli* O111:B4, L4391), adenosine triphosphate (ATP) (A6419), Tween-20 (P1379), dimethyl sulfoxide (DMSO) (D8418) and DL-dithiothreitol (DTT) (D0632) were purchased from Sigma-Aldrich (St. Louis, MO, USA). Nigericin (tlrl-nig, Purity: ≥98.0%) powder was bought from InvivoGen (San Diego, CA, USA), which was dissolved in ethanol. Dulbecco’s Modiffed Eagle’s Medium (DMEM) with high glucose (C11995500BT), RPMI Medium 1640 basic (C11875500BT), β-mercaptoethanol (21985-023), fetal bovine serum (FBS, 10099141C), 100 × streptomycin/penicillin (15140122) were bought from Invitrogen/Gibco (Carlsbad, CA, USA). Phorbol 12-myristate 13-acetate (PMA) (S1819), Co-IP lysis buffer (P0013), JC-1(C2005), Cell Mitochondria Isolation Kit(C3601M), Quick Genotyping Assay Kit for Mouse Tail (D7283S), Reactive oxygen species (ROS) assay kit (S0033S) and phenylmethanesulfonyl fluoride (PMSF) (ST505) were obtained from Beyotime (Shanghai, China). Specific antibodies against ASC (#67824), ASC-AlexaFluor647 conjugated (#23640), β-actin (#3700), cleaved caspase-3 (#9664), cleaved caspase-8 (#8592), caspase-9 (#9508), IL-1β (#12242), PARP (#9532), NLRP3 (#15101), TOMM20 (#42406), α-Tubulin (#3873), horseradish peroxidase (HRP)-conjugated horse-anti mouse IgG (#7076), HRP-conjugated goat-anti-rabbit IgG (#7074) mouse were purchased from Cell Signaling Technology (Danvers, MA, USA). Antibodies against pro-caspase1 + p10 + p12 (ab179515), GSDMD (ab209845), GSDME (ab215191) and pro-caspase-8 (ab108333) were bought from Abcam (Cambridge, UK). Antibodies against NLRP3 (AG-20B-0014) was purchased from Adipogen AG (Liestal, Switzerland). Antibodies against ASC (B-3) (sc-514414) was purchased from Santa Cruz Biotechnology (Dallas, Texas, USA).

### 2.2 Animals

C57BL/6J mice (WT) aged 6–8 weeks were obtained from GemPharmatech (Nanjing, China). Heterozygous *caspase-1^+/−^* mice on a C57BL/6J background were purchased from Cyagen Biosciences (Guangzhou, China). The *caspase-1* ^−/−^, and WT littermates used in this study were generated in-house by breeding heterozygous parents (5th to 9th generations). Mice with caspase-1 deletion exhibited no apparent physiological defects, and their size, body weight, and appearance were comparable to those of WT littermates. Genotyping was conducted using PCR-based methods with the Quick Genotyping Assay Kit for Mouse Ears (D7283, Beyotime; Shanghai, China), following the manufacturer’s protocol. All animals were housed at 25 °C under a 12-h light/dark cycle with ad libitum access to food and water, and were acclimatized for at least one week prior to experiments. All experimental procedures were approved by the Ethical Institutional Animal Care and Use Committee of Jinan University (EIACUC-JNU), Guangzhou, China.

### 2.3 THP-1 cells

Human THP-1 WT cells (kindly provided by Dr. Yao Wang of Sun Yat-sen University, Guangzhou, China) and THP-1 caspase-1-deficient cells (kindly provided by Professor Jun Cui in Sun-yat Sen University) were cultured in RPMI 1640 medium supplemented with 10% FBS and antibiotics. THP-1 cells were differentiated into macrophages incubation with 100 nM of PMA for 3 h (17).

### 2.4 RAW 264.7 cells

RAW 264.7 cells were bought from Kunming cell bank, CAS (Kunming, China). The cells were maintained in complete DMEM medium (containing 10% FBS, 1% penicillin and streptomycin) and cultured at 37 °C in a humidified incubator with 5% CO_2_.

### 2.5 Bone marrow-derived macrophage differentiation

BMDMs were differentiated from mouse bone marrow cells as described previously (18). Briefly, WT and *caspase-1^−/−^* C57BL/6J mice were sacrificed by cervical dislocation and sterilized with 75% ethanol. The bone marrow cells in hind femora and tibias were flushed out by 10 mL sterile 1×PBS and separated from the mixture by centrifugation at 1500 rpm for 5 min at 4 °C. Then the bone marrow cells were re-suspended in BM-Mac medium which is composed of 80% complete DMEM medium and 20% M-CSF-conditioned medium from L-929 cells, and cultured in the 10-cm petri dish with 10 mL BM-Mac medium at 37 °C in a humidified incubator of 5% CO_2_. On the third day of cell culture, half volume of fresh medium (5 mL/dish) was added and let them differentiate into BMDMs after 6 d. The cells were then collected by using cell-scraper and cultured in 6-well plates at 1.6 × 10^6^ cells/well (1.7 mL) or 24-well plates at 2.5 × 10^5^ cells/well (0.5 mL) with complete DMEM medium. The cells were ready for experiments after overnight incubation.

### 2.6 Cell treatment

WT BMDMs were primed with LPS (500 ng/mL) for 4 h and stimulated with ATP (3 mM) or nigericin (5 μM) for 0.5 h or 1 h to induce NLRP3 inflammasome activation and pyroptosis, respectively. For delayed NLRP3 activator stimulation assays, WT BMDMs were treated with LPS (200 ng/mL) for 4 h, washed, and cultured in fresh medium (without LPS) for 12 h (the resting step), and then were treated with or without the same dose of LPS for 4 h again. After that, the cells were stimulated with ATP (3 mM) for 0.5 h; or alternatively, cells were primed with LPS (500 ng/mL) for 4 h, washed and cultured in fresh medium. Nigericin (5 μM) was added either immediately or 12 h later for 0.5 h. For caspase-1 inhibitor assays, BMDMs, PMA-differentiated THP-1 cells (WT or *caspase-1*-deficient) or RAW264.7 cells (lacking ASC expression) were incubated with LPS (500 ng/mL) for 4 h, pre-treated with VX-765 (at indicated concentrations) or vehicle for 1 h, and then stimulation with nigericin (5 μM for BMDMs and 10 μM for THP-1 cells) for specified durations.

To prepare a positive control (PC) sample for Western blotting, BMDMs were treated with 5Z-7-oxozeaenol (TAK1 inhibitor) plus LPS to induce PANoptosis (concurrent Pyroptosis, Apoptosis, and Necroptosis) as previously described (19).

### 2.7 Cell death assay

Cell death was measured by PI incorporation assay or lactate dehydrogenase (LDH) release assay. Briefly, after indicated treatment, PI (2 µg/mL) and Hoechst 33342 (5 µg/mL) solutions were added into medium to stain the dying cells and the nuclei, respectively. Apoptotic cells are detected by using Annexin V-EGFP Apoptosis Detection Kit (C1062M, Beyotime). Among them, Annexin-V-EGFP^+^PI^—^ indicates early apoptotic cells, while Annexin-V-EGFP^+^PI^+^ indicates late apoptotic or necrotic cells. The stained cells were observed by live imaging using Zeiss Axio Observer D1 microscope equipped with a Zeiss LD Plan-Neofluar 20×0.4 Korr M27 objective lens (Carl Zeiss MicroImaging GmbH, Go¨ttingen, Germany). Fluorescence images were captured with the Zeiss AxioCam MR R3 cooled CCD camera controlled with ZEN software (Carl Zeiss). For LDH release assay, the lytic cells were evaluated by using the CytoTox 96 non-radioactive cytotoxicity assay kit (G1780, Promega) according to instructions of the manufacturer.

### 2.8 Western blot analysis

Western blotting was performed as previously described (18). Briefly, whole cell lysates were separated by sodium dodecyl sulfate–polyacrylamide gel electrophoresis (SDS-PAGE) and electrotransferred to PVDF membranes (03010040001; Roche Diagnostics GmbH, Mannheim, Germany), which were blocked by blocking buffer for 1 h once the electro-transfer had been completed. Then, the membrane was incubated with primary antibody at 4 °C overnight, followed by incubation with appropriate HRP-conjugated secondary antibody. The target bands were visualized with an enhanced chemiluminescence kit (BeyoECL Plus; Beyotime, Shanghai, China) and captured on X-Ray films. The results were recorded by FluorChem 8000 imaging system (AlphaInnotech, San Leandro, CA, USA) and analyzed by AlphaEaseFC 4.0 software (AlphaInnotech).

### 2.9 Precipitation of soluble proteins

After the cells were treated, the cell culture supernatant was collected in a 1.5 ml Eppendorf tube, centrifuged at 300 *×g* for 5 min, and the supernatant was transferred to a new 1.5 mL Eppendorf tube. The protein in the cell culture supernatant was precipitated with 10% sodium deoxycholate and trichloroacetic acid. Overnight precipitation at 4 °C and centrifugation at 14,000 rpm for 30 min; The supernatant was discarded, 800 μL acetone pre-cooled at -20 °C was added, ultrasonic dissolution and precipitation were allowed to stand for 10 min, centrifuge at 14,000 rpm for 5 min, and acetone was repeatedly washed and precipitated for 3 times. After the acetone was completely volatilized, 2×SDS-PAGE loading buffer was added, the protein precipitate was completely dissolved by full shock, and the solution was boiled in boiling water for 5 min and stored at -80 °C.

### 2.10 Immunofluorescence microscopy

Immunofluorescence analysis as described previously (20), in short, BMDMs were performed in a glass-bottomed confocal dish (#801002; NEST, Wuxi, China), 1.5×10^5^ cells/well, 37 °C overnight. After the indicated treatment, the cells were fixed with 4% paraformaldehyde for 15 min and permeated with 2 mL cold methanol at -20 ˚C for 10 min. After penetration, the cells are blocked with a blocking buffer. After overnight incubation with the specified antibody, incubated cells were incubated with CF568 anti-Rabbit IgG (1:500) and CF488 anti-mouse IgG (1:500) at room temperature for 1 h, then coupled with anti-ASC-AlexaFluor647. The nucleus was then displayed with Hoechst 33342 solution (5 μg/mL).

### 2.11 Detection of cellular ROS

Reactive oxygen species (ROS) assay kit (S0033, Beyotime) was adopted to detect the total intracellular ROS produced in living cells according to the supplier’s instructions. The fluorescence of intracellular DCFH-DA was observed with Axio Observer D1 microscope (Carl Zeiss) and images were captured. The average fluorescence intensity was quantified by ZEN software (Carl Zeiss).

### 2.12 Mitochondrial membrane potential analysis

After the cells were treated, JC-1 working solution (5 μg/mL) was added for evaluating mitochondrial membrane potential (MMP), and the cells were incubated at 37 °C without light for 30 min, and then washed twice with warm serum-free DMEM or staining buffer. Stained cells were observed by live imaging using Zeiss Axio Observer D1 microscope. Once entering cells, JC-1 becomes aggregates (red fluorescence) in intact mitochondria, but becomes monomers (green fluorescence) if the mitochondrial membrane is damaged (loss of MMP). Data were acquired and analyzed by using the software of ZEN (Carl Zeiss). Image analysis of fluorescence intensity was performed using ImageJ, while statistical analysis was conducted with GraphPad Prism 6.0. Loss of MMP was presented as the ratio of cells with mitochondrial monomer.

### 2.13 Mitochondrial isolation

Isolation of mitochondria was performed using Beyotime’s Cell Mitochondria Isolation Kit (C3601), according to the instruction of the manufacturer. The purity of isolated mitochondria is monitored by Western blotting, with TOMM20 serving as a mitochondrion marker as it is distributed in the outer membrane of mitochondria, and α-Tubulin, a cytoskeleton protein, as a cytosol marker. The distributions (cytosol or mitochondrion) and levels of GSDMD and Caspase-1 were evaluated by Western blotting.

### 2.15 Statistical analysis

Each experiment was performed three times independently. Data were expressed as mean ± standard deviation (SD) and analyzed for statistical significance using GraphPad Prism 6.0 (GraphPad Software Inc, San Diego, CA, USA). One-way analysis of variance (ANOVA) followed by Tukey *post hoc* test was used to analyze the statistical significance among multiple groups. Significance was defined as follows: *P < 0.05, **P < 0.01, and ***P < 0.001.

## 3 Results

### 3.1 Delayed nigericin stimulation arouses weakened caspase-1 activation but increases caspase-8 activation in LPS-primed macrophages

**Figure 1:**
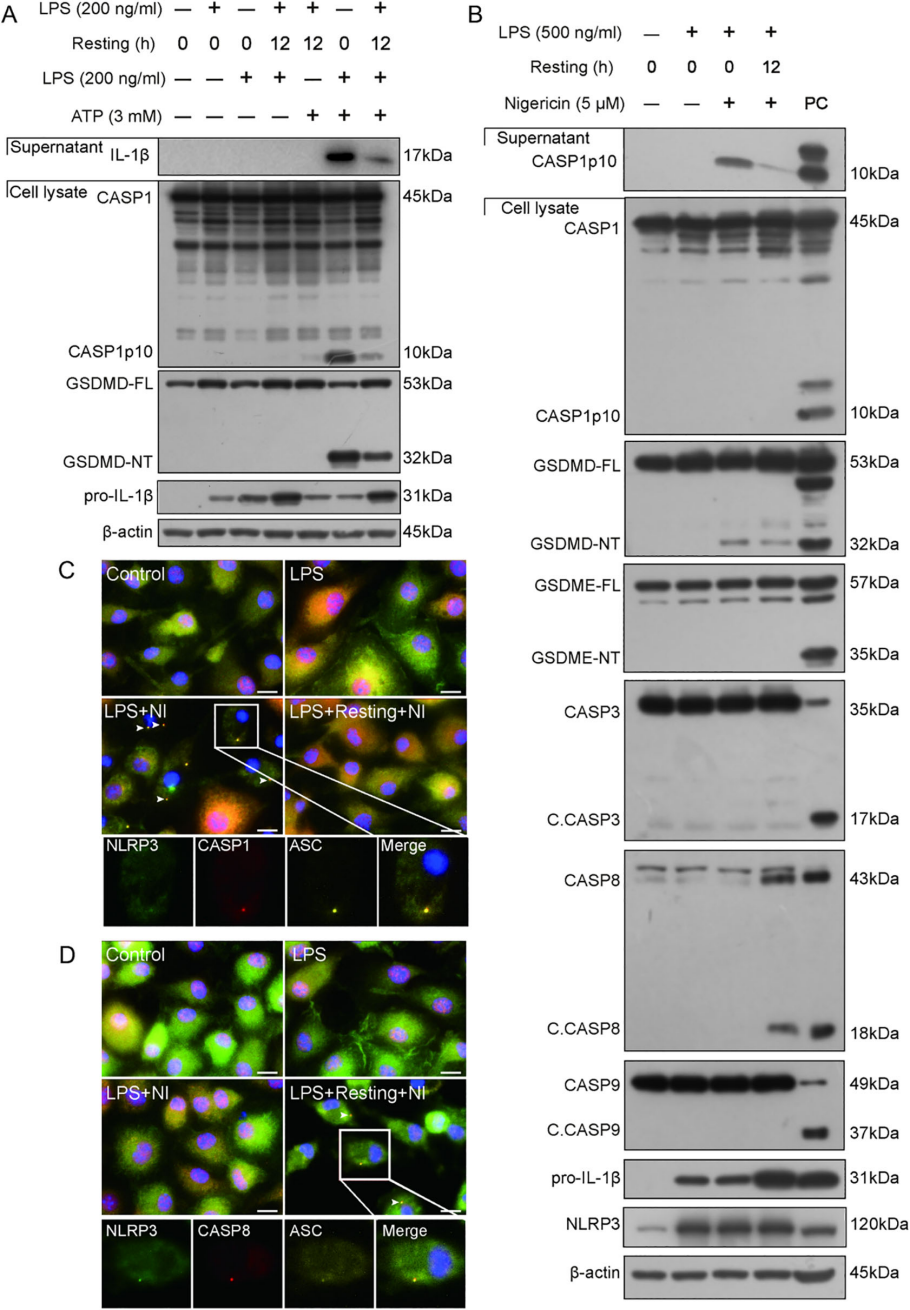
Delayed stimulation of NLRP3 activator attenuates NLRP3 inflammasome activation and induces the formation of NLRP3/ASC platform to trigger apoptotic caspase activation. **(A, B)** Wild-type (WT) BMDMs were treated with LPS, resting for indicated time length, and then stimulated with ATP **(A)** or nigericin **(B)** as described in Section 2.6. Proteins in the cell lysates were analyzed by Western blotting. **(C, D)** Immunofluorescence images show the subcellular distribution of ASC, caspase-1, and NLRP3 **(C)**, or ASC, caspase-8, and NLRP3 **(D)**. Nuclei were revealed by Hoechst 33342 (blue). Scale bar, 20 μm. Insets were shown at the bottom with separate images and their merge. C. CASP, cleaved CASP; GSDMD-FL, full-length GSDMD; GSDMD-NT, N-terminal GSDMD fragment; GSDME-FL, full-length GSDME; GSDME-NT, N-terminal GSDME; resting, culturing cells in fresh complete medium (treatment) after being washed with serum-free DMEM; PC, positive control.

In this study, we first explored the response of LPS-tolerant macrophages to a triggering signal of NLRP3 inflammasome (nigericin or ATP). To address this, we established an LPS tolerant cellular model by stimulating BMDMs with double 4-h LPS treatments (with a 12-h interval between them). The results showed that LPS tolerant macrophages were less sensitive to ATP stimulation in view of the NLRP3 inflammasome activation (as evidenced by GSDMD-NT and caspase-1p10) (**Figure 1A**), showing a reduced response of LPS tolerant macrophages, although the expression of pro-IL-1β (due to NF-κB activation) could still be increased in response to the second LPS treatment. Similarly, a 12-h interval between the LPS (only once) and nigericin treatments, compared to immediate stimulation with nigericin after LPS priming, induced a reduced NLRP3 inflammasome and caspase-1 activation, but an increased caspase-8 activation (**Figure 1B**), suggesting activation of an alternative pathway. Immunofluorescence microscopy was used to observe the distribution of NLRP3, caspase-1, and caspase-8 in LPS-primed WT BMDMs that were stimulated with nigericin (immediately or delayed). In the cells immediately stimulated with nigericin, NLRP3, ASC, and caspase-1 were co-localized and formed a speck (indicating the assembly of NLRP3 inflammasome) (**Figure 1C**). But in those stimulated with nigericin 12-h later, no NLRP3 inflammasome containing caspase-1 were observed; instead, puncta of NLRP3, ASC, and caspase-8 were co-localized (**Figure 1D**), suggesting that a delayed triggering signal leads to caspase-8 recruitment to the NLRP3/ASC platform, which could trigger apoptotic caspase activation.

### 3.2 Nigericin activates apoptotic caspases in caspase-1-deficient macrophages

**Figure 2:**
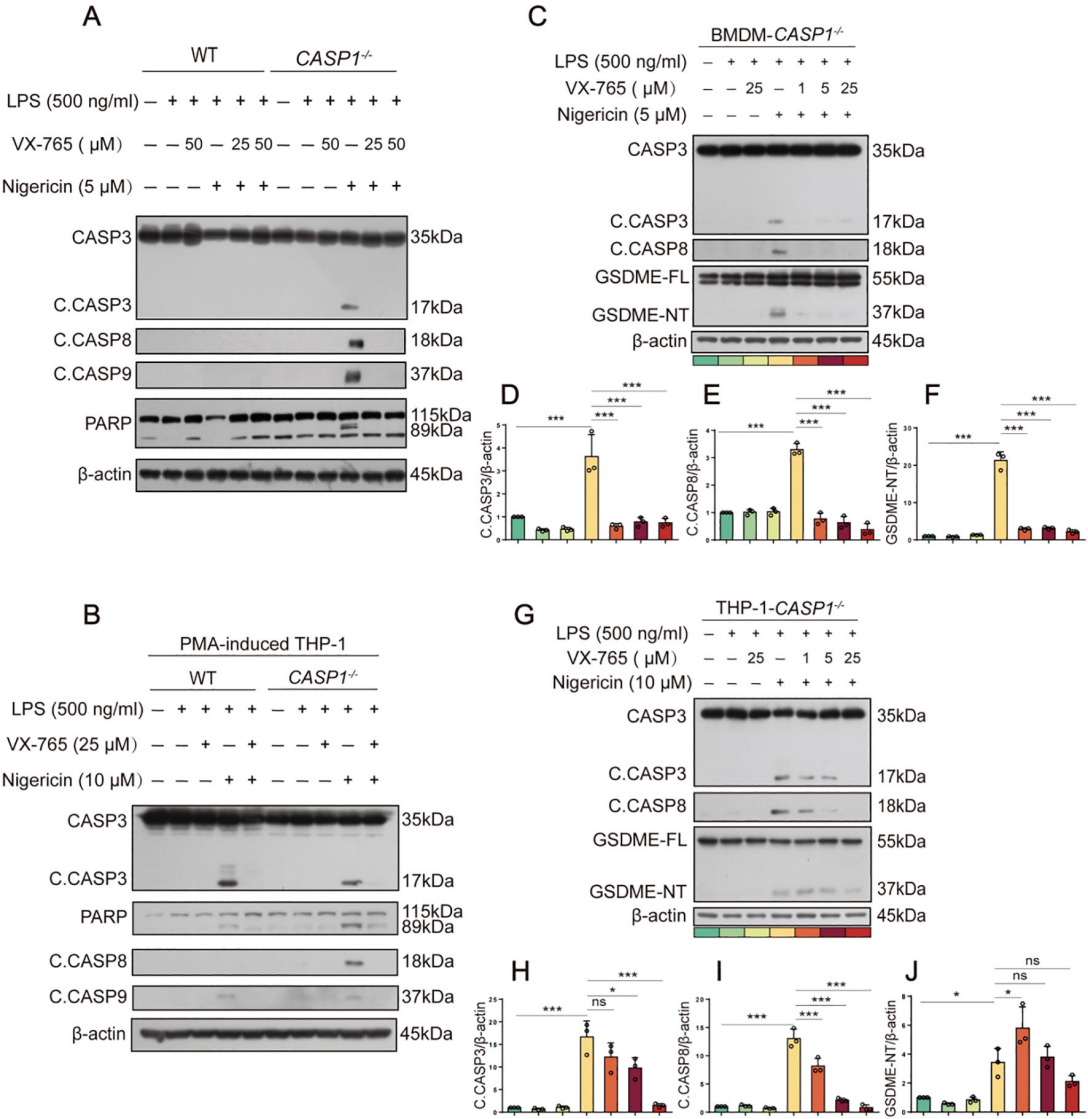
NLRP3 activators induce apoptotic caspase activation in caspase-1-deficient macrophages. **(A, B, C, G)** WT or caspase-1-deficient BMDMs or PMA-differentiated THP-1 cells were primed with LPS for 4 h, treated with caspase-1 inhibitor VX-765 for 1 h, and then stimulated with nigericin for 1 h as described in Section 2.6. Proteins in the cell lysates were analyzed by Western blotting. Relative gray values of C. CASP8, C. CASP3, GSDME-NT blots were quantified [**(D-F)** corresponding to **(C)**; **(H-J)** to **(G)**]. Data are shown as mean ± SD (*n* = 3). *P < 0.05; ***P < 0.001; ns, not significant. GSDME-FL, full-length GSDME; GSDME-NT, N-terminal fragment of GSDME; C. CASP, cleaved CASP.

Although the above-mentioned results showed that nigericin induced caspase-8 activation in LPS-tolerant macrophages, it is unclear whether caspase-1 is required for the NLRP3/ASC platform to recruit caspase-8 and other apoptotic caspases. Thus, we evaluated whether nigericin could induce apoptotic caspase activation in caspase-1-deficient macrophages. Western blotting corroborated that apoptotic caspases, including caspase-8, -9, and -3 (indicative of apoptosis), were all activated in caspase-1-deficient BMDMs but not in WT cells (**Figure 2A**). Similar results of caspase-8 activation were achieved using caspase-1-deficient THP-1 macrophages (**Figure 2B**). These results suggest that NLRP3 activators prefer activating caspase-1 to caspase-8 and other apoptotic caspases, but when caspase-1 is deficient, the NLRP3 activators activate apoptotic caspases in macrophages.

Unexpectedly, caspase-1 inhibitor VX-765 could also inhibit the activation of apoptotic caspases (caspase-8, -9, -3, *etc.*) in both BMDMs and THP-1 macrophages (**Figures 2C, G**). Lower concentrations of VX-765 (1-25 μM), which inhibits caspase-1 activation, still inhibited the activation of caspase-8, -9, and -3 upon nigericin stimulation in a dose-dependent manner (**Figures 2D-F, H-J**). Consequently, nigericin induced the cleavage of GSDME by activative caspase-3 in caspase-1-deficient BMDMs and THP-1 macrophages. This effect could be mitigated dose-dependently by VX-765 (**Figure 2**). Thus, VX-765 shows a property of pan-caspase inhibitor, at least in circumstances of NLRP3 activator stimulation.

### 3.3 NLRP3/ASC speck is co-localized with apoptotic caspases in caspase-1-deficient macrophages upon the stimulation with NLRP3 activators

**Figure 3:**
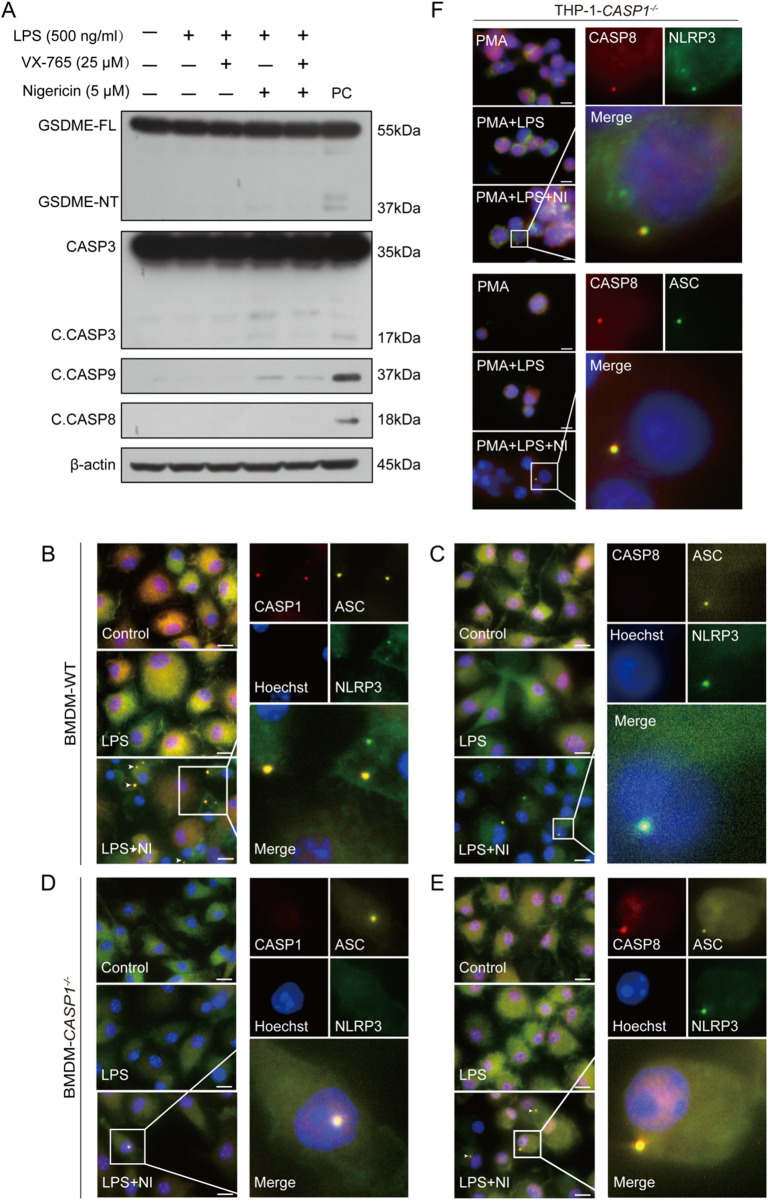
ASC mediates the formation of a platform and the activation of caspase-8 in caspase-1-deficient macrophages upon NLRP3 stimulation. RAW 264.7 cells **(A)** or WT and caspase-1-deficient mouse BMDMs **(B-E)** were treated as described in Section 2.6. Representative immunofluorescence images show fluorescence staining of ASC, caspase-1 and NLRP3 **(B, D)** and ASC, caspase-8 and NLRP3 **(C**, **E)**. Nuclei were stained with Hoechst 33342 (blue). **(F)** PMA-differentiated THP-1 caspase-1-deficient macrophages were primed with LPS and stimulated with nigericin as described in Section 2.6. Representative immunofluorescence images show fluorescence staining of caspase-8 and NLRP3 or ASC. The images were captured respectively and merged. Scale bars, 20 µm. GSDME-FL, full-length GSDME; GSDME-NT, N-terminal GSDME;C.CASP, cleaved CASP; PC, positive control.

Previous studies have reported that the AIM2/ASC complex can become a platform for caspase-8 activation when caspase-1 is deficient (21). It is proposed that ASC serves as an adaptor between NLRP3 and caspase-8. Indeed, our results showed that nigericin could not induce the activation of caspase-8 in ASC-deficient RAW 264.7 cells, although it activated caspase-9 weakly. Consequently, cleaved caspase-3 and GSDME fragments upon nigericin treatment were quite weak in RAW 264.7 cells as compared to their respective full-length proteins (**Figure 3A**).

**Figure 4:**
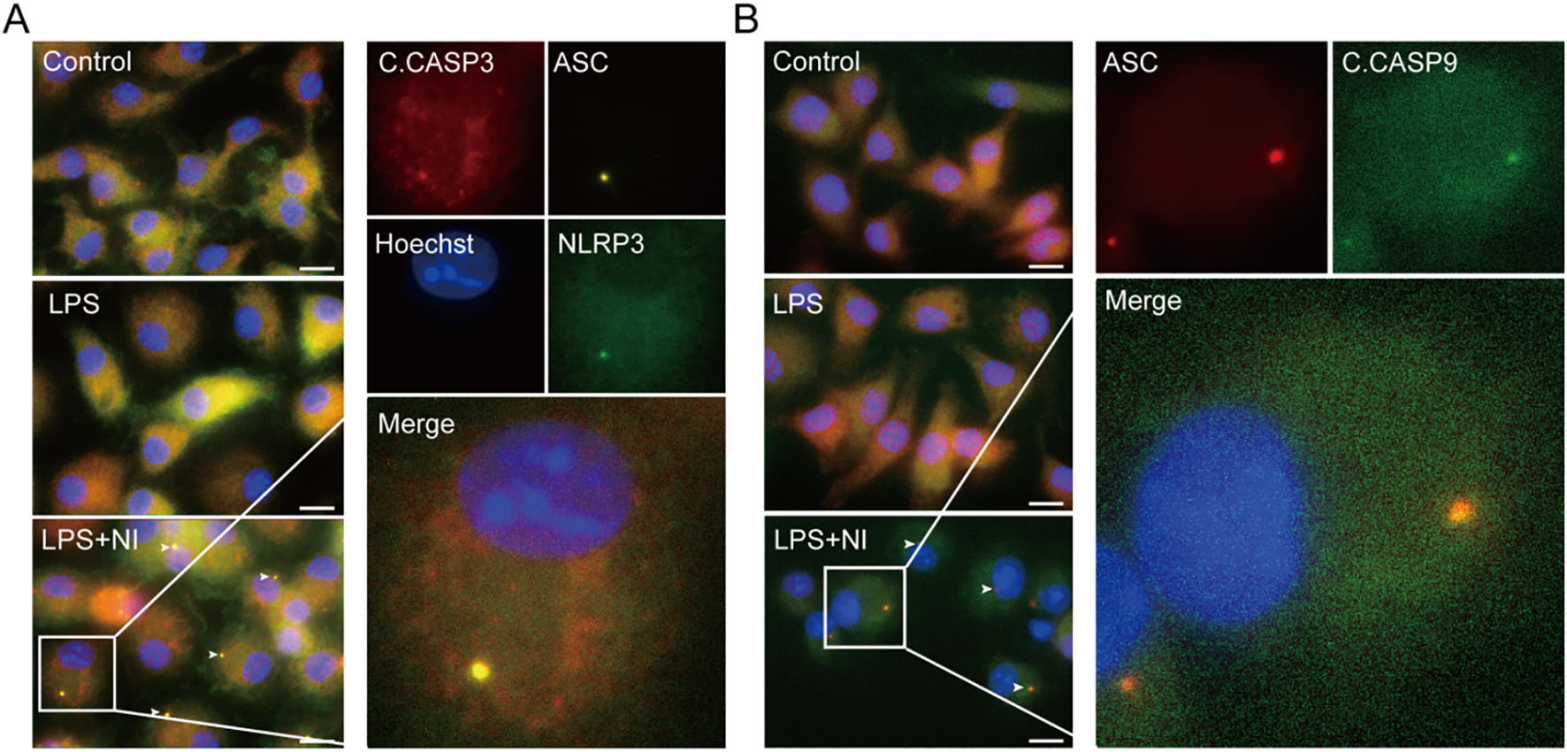
NLRP3 activators can induce the formation of NLRP3/ASC platform for the activation of apoptotic caspases in caspase-1-deficient macrophages. Caspase-1-deficient BMDMs were primed with LPS and then stimulated with nigericin. Immunofluorescence images show the fluorescent staining of ASC, C. CASP3 and NLRP3 **(A)**, the fluorescent staining of ASC and C. CASP9 **(B)**. Nuclei were stained with Hoechst 33342 (blue). Images were captured separately and merged. Scale bar, 20 μm. C.CASP, cleaved CASP; NI, nigericin.

Next, we explored whether NLRP3, ASC, and apoptotic caspases could form a complex upon nigericin stimulation in WT and caspase-1-deficient macrophages by immunofluorescence microscopy. The images showed that NLRP3, ASC, and caspase-1, rather than caspase-8, were co-localized with each other in WT BMDMs (**Figures 3B, C**); but in their caspase-1-deficient counterparts, NLRP3, ASC, and instead caspase-8 were co-localized to form a speck together (**Figures 3D, E**). Similarly, in caspase-1-deficient THP-1 cells, caspase-8 was observed to form a speck with ASC and NLRP3, respectively (**Figure 3F**). The co-localization of NLRP3, ASC, and caspase-3 or caspase-9 (using anti-C. CASP3 and anti-C. CASP9 antibodies) were also observed in caspase-1-deficient cells upon the stimulation with nigericin (**Figure 4**), suggesting that the NLRP3/ASC complex can serve as a platform for apoptotic caspases activation when caspase-1 is deficient.

### 3.4 Nigericin induced GSDMD-mediated pyroptosis in WT macrophages but not in caspase-1-deficient macrophages

**Figure 5:**
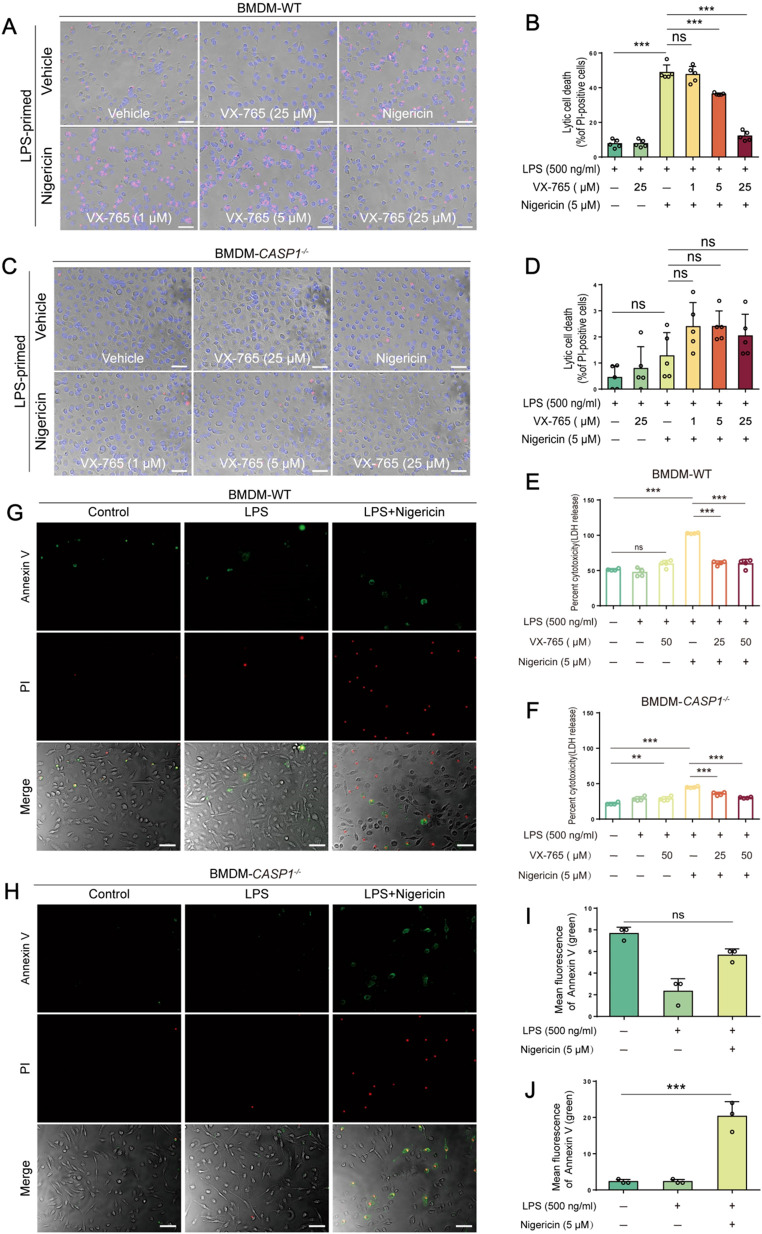
NLRP3 activators induce GSDMD-mediated pyroptosis in WT macrophages but not in caspase-1-deficient ones. WT **(A, B)** and caspase-1-deficient **(C, D)** mouse BMDMs were first primed with LPS, and then pretreated with or without VX-765 followed by nigericin stimulation. Cell death was assayed by propidium iodide (PI) (red) incorporation **(A, C)** (n=5) and lactate dehydrogenase (LDH) release **(E, F)** (n=4). The percentage of cell death is defined as PI-positive cells to all ones (Hoechst 33342-positive) in 5 randomly-chosen fields **(B, D)**. **(G-J)** Representative images showing bright-field images combined with green fluorescence (Annexin-V-positive, indicating early apoptotic cells) and red fluorescence (PI-positive, indicative of late apoptotic or necrotic cells). Data are shown as the mean ± SD (n ≧ 3). **P < 0.01; ***P < 0.001; ns, not significant.

Unlike their WT counterparts, caspase-1-deficient BMDMs did not exhibit typical morphological features of pyroptosis, such as cell membrane bubbling and eventual membrane rupture, upon nigericin stimulation. Instead, most cells appeared normal or became rounded and shrunken while maintaining intact plasma membranes, consistent with an apoptotic morphology (**Figures 5A, C**). PI incorporation (**Figures 5A, B**) and LDH release (**Figure 5E**) assays indicated that nigericin induced lytic cell death in WT macrophages, which could be inhibited by VX-765 in a dose-dependent manner. In contrast, caspase-1-deficient cells showed less PI incorporation (**Figures 5C, D**) and LDH release (**Figure 5F**) compared to their WT counterparts upon the same dose of nigericin stimulation. Consistent with the activation of apoptotic caspases (**Figure 2**), nigericin induced a higher proportion of Annexin-V^+^ (an early apoptosis marker) cells in caspase-1-deficient BMDMs compared to WT cells (**Figures 5G-J**), suggesting differential cell death signaling pathways in these two types of cells.

**Figure 6:**
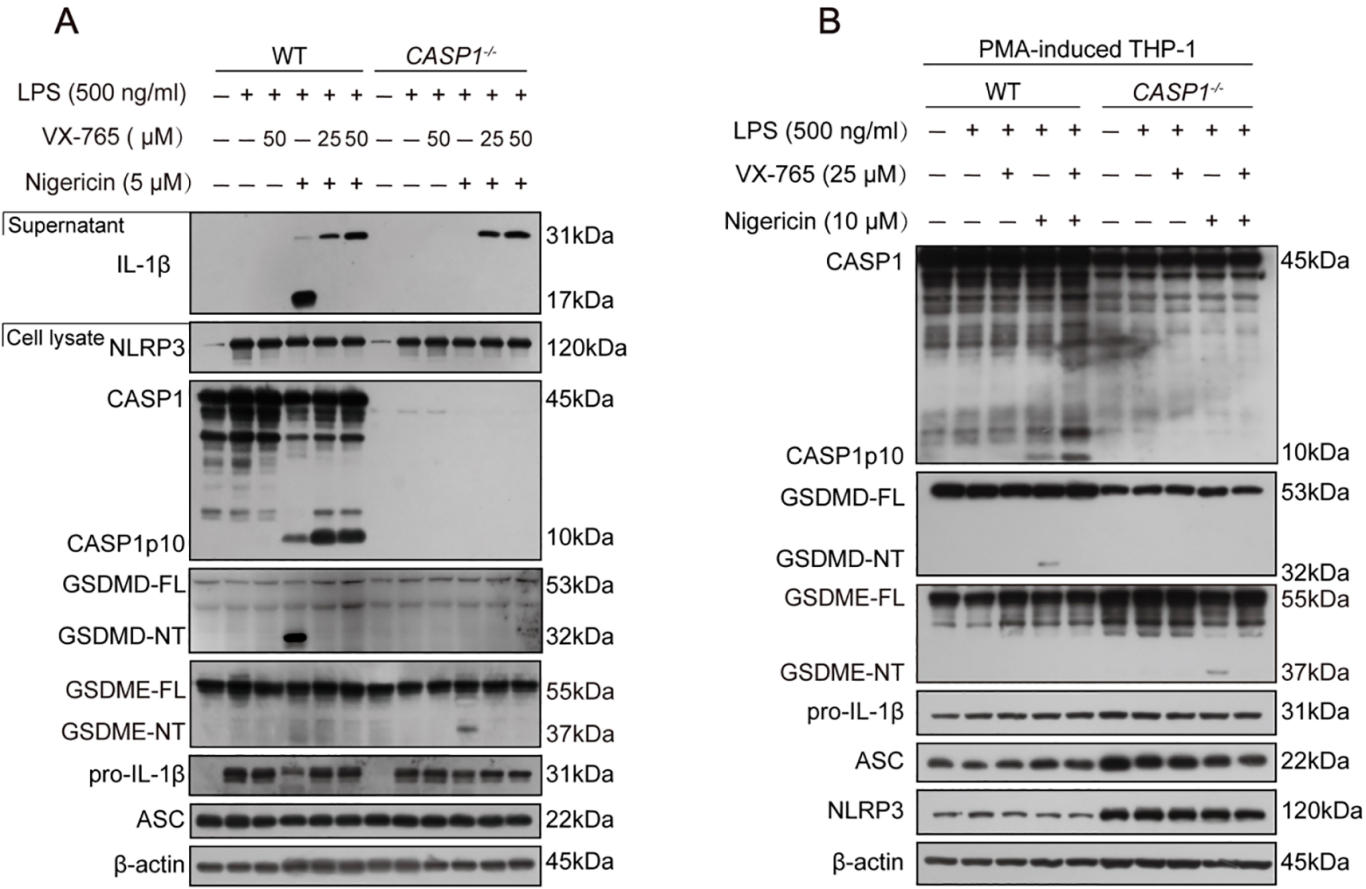
NLRP3 activators induce pyroptosis in WT macrophages but not in caspase-1-deficient ones. **(A)** WT and caspase-1-deficient mouse BMDMs were first primed with LPS, and then pretreated with or without VX-765 followed by nigericin stimulation for 0.5 h. **(B)** PMA-differentiated WT and caspase-1-deficient THP-1 macrophages were primed with LPS, and then treated with VX-765 followed by nigericin stimulation. Proteins in the cell lysates were analyzed by Western blotting. GSDMD-FL, full-length GSDMD; GSDMD-NT, N-terminal GSDMD fragment; WT, wild-type.

Characterizing the lytic cell death in the above-mentioned cells, Western blotting corroborated that LPS plus nigericin induced the activation of caspase-1 (as indicated by caspase-1p10 fragment), leading to the release of IL-1β and cleavage of GSDMD (indicative of pyroptosis), in both WT BMDMs and THP-1 macrophages, but not in their caspase-1-deficient counterparts, respectively. As expected, VX-765 inhibited the release of IL-1β and the cleavage of GSDMD in these WT macrophages (**Figures 6A, B**). As indicated above, nigericin could induce weak GSDME cleavage by caspase-3 in caspase-1-deficient macrophages instead of WT ones (**Figure 2**, **Figure 6**), leading to secondary necrosis (**Figures 5C, H**). These results indicate that nigericin induces GSDMD-mediated pyroptosis in WT macrophages while inducing apoptosis and GSDME-mediated secondary necrosis in caspase-1-deficient cells.

### 3.5 NLRP3 activators induce mitochondrial damage in both WT and caspase-1-deficient macrophages

**Figure 7:**
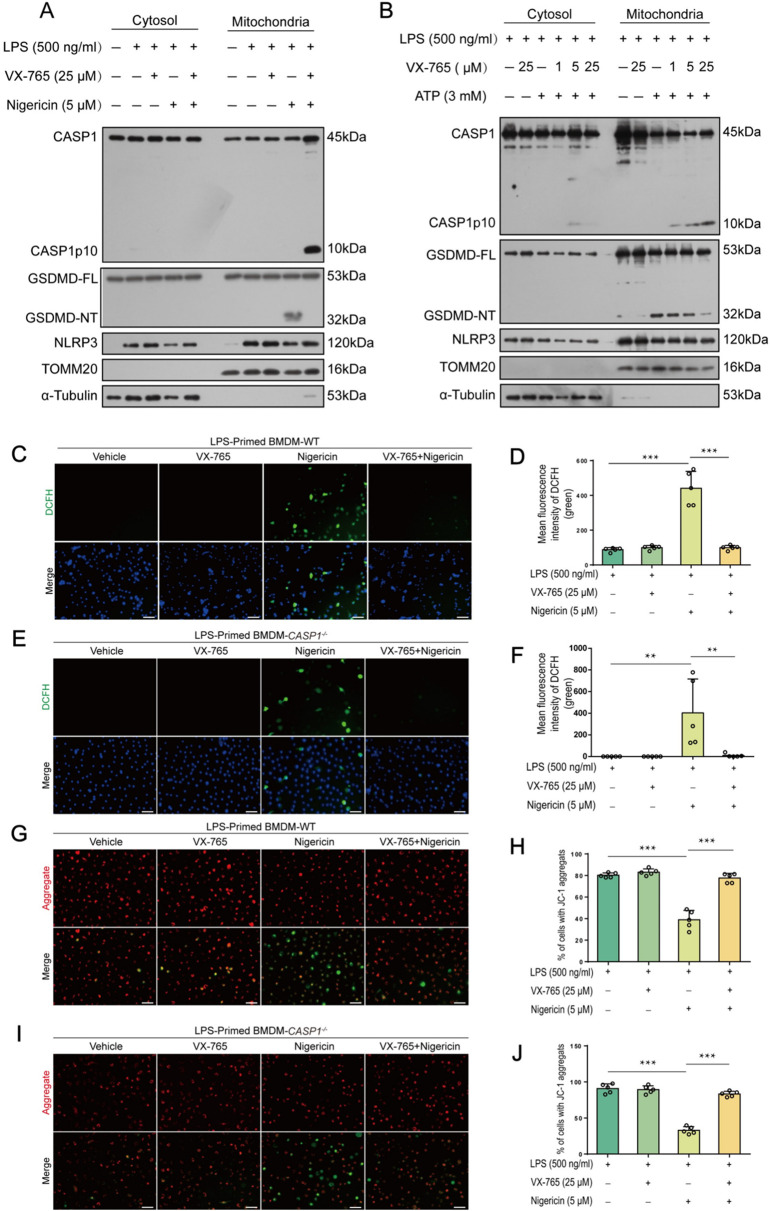
VX-765 alleviates mitochondrial damage induced by ATP and nigericin in both wild-type (WT) and caspase-1-deficient macrophage. **(A, B)** WT mouse BMDMs were first primed with LPS, pretreated with VX-765, and stimulated nigericin **(A)** or ATP **(B)**, as described in Section 2.6. Western blot analysis of cytosolic and mitochondrial proteins was performed after mitochondrial and cytosol isolation, with TOMM20 serving as a mitochondrial marker while α-Tubulin as a cytosol marker. **(C, E)** WT and caspase-1-deficient BMDMs were treated with LPS and nigericin as **(A)**. Reactive oxygen species (ROS) in cells were detected by using 2’,7’-dichlorodihydrofluorescein diacetate (DCFH-DA) and observed by fluorescence microscopy. **(D, F)** Histograms showing quantitative analyses of **(C, E)**. **(G, I)** JC-1 was used to assay the mitochondrial membrane potential in the cells. **(H, J)** Quantitative analyses of ratios of cells with JC-1 aggregates (red fluorescence). Data are shown as mean ± SD (*n* = 5). Scale bars, 50 μm. **P < 0.01; ***P < 0.001. GSDMD-FL, full-length GSDMD; GSDMD-NT, N-terminal fragment of GSDMD.

Consistent with previous studies that NLRP3 activators can induce mitochondrial damage (2, 22), an upstream event that triggers NLRP3 activation (23–25), our Western blot data showed that nigericin- and ATP-induced GSDMD-NT fragments were present in the mitochondrial parts, while VX-765 dose-dependently reduced their levels (**Figures 7A, B**). Consistently, nigericin increased ROS in WT macrophages, which could be inhibited by VX-765 (**Figures 7C, D**). Interestingly, nigericin also increased ROS in caspase-1-dificient BMDMs (**Figures 7E, F**). It induced the loss of MMP in both caspase-1-deficient and WT macrophages, but could be rescued by VX-765 treatment (**Figures 7G-J**). These results indicate that NLRP3 activators induce mitochondrial damage in both WT and caspase-1-deficient macrophages, suggesting mitochondrial injury being an upstream event in this process.

## 4 Discussion

Danger signal-induced inflammasome activation drives caspase-1-mediated cytokine secretion (e.g., IL-1β/IL-18) and lytic cell death (pyroptosis), which are critical for host defense against pathogenic infections or tissue damage (26). In WT macrophages, caspase-1 activation requires both priming (such as LPS) and triggering (*e.g.*, ATP or nigericin) signals. However, our data revealed that caspase-8 could be alternatively activated on the NLRP3/ASC platform in the absence of caspase-1. This is consistent with prior reports of caspase-8-mediated pro-IL-1β processing in dendritic cells during *C. albicans* infection (27). The *Pseudomonas* effector ExoS inhibits caspase-1 activation, thereby reducing the death rate of infected cells (28). Intriguingly, *P. aeruginosa* can induce the activation of caspase-3/-7 in an ASC-dependent manner if caspase-1 was absent (29). These studies demonstrate that inflammasomes may serve as platforms for divergent caspase pathways.

Caspase-8 can serve as a switch of various regulated cell death (30). It contains two tandem death effector domains (DEDs) at its N-terminus and a catalytic domain at its C-terminus. Through oligomerization mediated by its DEDs, caspase-8 is activated, and it not only cleaves Poly (ADP-ribose) polymerase (PARP) family proteins (*e.g.*, PARP-2), but also activates downstream caspase-3, thereby initiating the apoptotic cascade (31–33). It also suppresses necroptosis through forming a complex with FADD and cFLIP, which antagonizes the RIPK1-RIPK3-MLKL (necroptotic) pathway (34–36). Similarly, *Burkholderia pseudomallei* infection induces NLRC4/caspase-1 activation at an early stage, but causes a delayed activation of apoptotic caspases (caspase-7, -9) in an NLRC4- and caspase-1-dependent manner (37), though the switch mechanism remains unclear.

Our data suggest that a delayed inflammasome triggering (*e.g*., late nigericin stimulation) promotes caspase-8/-9/-3 activation, particularly in caspase-1-deficient or LPS-tolerant macrophages. Both caspase-8 and -3 were observed to co-localize with NLRP3 and ASC, suggesting their activation on the NLRP3/ASC complex. ASC is an adaptor of NLRP3 inflammasome, which interacts with NLRP3 and caspase-1 through its PYD and CARD domains, respectively (38). ASC’s role as a scaffold for both inflammasome and apoptosome components is further highlighted in cells undergoing PANoptosis (concurrent activation of Pyroptosis, Apoptosis, and Necroptosis) during influenza A infection (39). ASC is proposed to organize the PANoptosome (40, 41), but how it coordinates pyroptotic (caspase-1) and apoptotic caspases remains unresolved. In WT cells, ASC appears to prefer binding caspase-1 to apoptotic caspases, ensuring preferably activating inflammasome pathway than apoptosome one. One possible explanation is that caspase-1 possesses a CARD domain for interacting with ASC, whereas caspase-8 and -3 lack this domain. However, some studies have indicated that oligomerized ASC can physically bind caspase-8 via ASC’s PYD, while Pyrin competitively interacts with ASC to inhibit caspase-8-mediated apoptosis (42). However, whether ASC interacts with caspase-8 in a similar manner in the setting of our study warrants more investigation.

In ASC-deficient macrophages (*e.g*., RAW 264.7 cells (43)), NLRP3 activators could not induce inflammasome formation and caspase-1 activation, nor could they induce caspase-8 activation (**Figure 3A**), further corroborating that caspase-8 was activated through interaction with ASC. In addition to mouse macrophages, we also recruited human THP-1-derived macrophages (differentiated with PMA). Unlike in mouse WT BMDMs, pro-IL-1β, ASC and NLRP3 were constitutively expressed in WT THP-1 macrophages without requiring LPS stimulation. However, it is unclear whether this was the cause for their varied sensitivities to pathogen infections and other inflammatory stimuli (44, 45). One phenomenon is that nigericin induced both pyroptotic caspase-1 and apoptotic caspase-3/-9 activation in WT THP-1 macrophages.

Although multiple studies have shown that caspase-8 can directly cleave pro-IL-1β into mature IL-1β in circumstances such as TNF-α-induced systemic inflammatory response syndrome (SIRS) (46, 47), mature IL-1β fragments were undetectable in caspase-1-deficient macrophages upon nigericin treatment (**Figure 6**). Notably, nigericin induced GSDME cleavage in caspase-1-deficient macrophages (**Figure 2**), leading to secondary necrosis (**Figure 5**). However, whether this was mediated by caspase-8 warrants more investigation.

The non-canonical activation of apoptotic caspases in caspase-1-deficient macrophages instead of WT ones was unlikely attributable to their differential NLRP3 expression, since NLRP3 expression was induced by LPS in both cell types. However, a direct comparison of their NLRP3 expression under synchronous culture conditions would provide more accurate evidence, which warrants future investigation.

Caspase-1 is activated by autocleavage of its precursor on inflammasomes. The levels of its p10 or p20 fragments in the cell lysates or supernatants (released from cells) can generally reflect the activity of caspase-1 in these cells, which can be inhibited by the specific inhibitor VX-765 (48, 49). This compound primarily crosses the blood-brain barrier, and has demonstrated potent effects against various inflammatory disorders such as Alzheimer’s disease (50–52). Supporting its anti-caspase-1 activity, VX-765 inhibited nigericin-induced cleavage of GSDMD and pro-IL-1β (**Figure 6**). Unexpectedly, VX-765 potently suppressed apoptotic caspases (caspase-8, -9, and -3) at a low concentration (1 μM), showing a pan-caspase inhibitor property. In the presence of VX-765, however, caspase-1p10 fragments induced by NLRP3 activators were accumulated in the cells (**Figures 6A, B**, **7A, B**). Further investigation revealed that these p10 fragments were trapped in mitochondria by VX-765, with the cause being unknown yet. This phenomenon suggests that the caspase-1p10 levels in cell lysates might not accurately reflect caspase-1 activity in the presence of VX-765.

Macrophage-derived IL-1β and IL-18 play pivotal roles in recruiting other immune cells, particularly adaptive ones, to the sites of infection (53). The maturation of these cytokines requires a secondary danger signal to trigger inflammasome activation (caspase-1 activation). Otherwise, chronic pathologies such as chronic hepatic diseases with liver fibrosis, cirrhosis, and portal hypertension may be developed due to anergy of inflammasome activation response (54). Our research reveals that the timing of a secondary signal (e.g., nigericin) critically determines caspase activation pathways in LPS-primed macrophages. Delayed nigericin stimulation suppresses caspase-1 while enhancing caspase-8 activation. Notably, LPS-tolerant macrophages fail to regain inflammasome activation upon repeated LPS stimulation. The predominance of caspase-8 (without releasing IL-1β and IL-18) reshapes inflammatory responses and cell death mechanisms during infection. How to overcome the anergy of inflammasome activation and avoid chronic inflammation due to delayed triggering signals warrants more investigation.

## 5 Conclusion

The NLRP3/ASC complex dynamically recruits caspases, with caspase-1 dominating under timely stimulation with NLRP3 activators but yielding to apoptotic caspases upon delayed stimulation or caspase-1 deficiency. Both caspase-1 and apoptotic caspases could be inhibited by VX-765 under such circumstances, which was once regarded as a caspase-1-specific inhibitor. Our data may partially explain the mechanism of inflammasome activation anergy in LPS-tolerant macrophages, a phenomenon linked to chronic inflammation.

## Data Availability

The original contributions presented in the study are included in the article/supplementary material. Further inquiries can be directed to the corresponding authors.
